# Advancing Age-Friendly Initiatives in Higher Education: Insights From CU Anschutz Medical Campus

**DOI:** 10.1093/geroni/igaf122.1934

**Published:** 2025-12-31

**Authors:** Jodi Waterhouse

**Affiliations:** CU Anschutz Medical Campus, Aurora, Colorado, United States

## Abstract

The successful implementation of Age-Friendly Work across higher education institutions hinges on the collaboration of diverse campus stakeholders, including supporters, decision-makers, doers, and creative thinkers. These key players are instrumental in helping the campus Age-Friendly Champion establish a robust foundation for age-friendly initiatives, identify impactful projects, and deliver results that foster an age-friendly campus culture. The CU Anschutz Medical Campus, one of only four medical campuses worldwide to receive the prestigious age-friendly designation, will present its journey in identifying key stakeholders and forming an effective Age-Friendly Task Force. This presentation will detail the composition of the Task Force and how it strategically aligned the three age-friendly university domains—Research and Innovation, Encore Work, and Intergenerational Learning—to integrate age-friendly principles throughout the campus. Attendees will gain insight into the framework developed by CU Anschutz Medical Campus to create, implement, deliver, and evaluate initiatives within each of these domains. The presentation will showcase specific projects that have been successfully executed, demonstrating how they support age-friendly principles and benefit visitors, faculty, staff, and students across all Schools and Colleges. Join us to learn how CU Anschutz Medical Campus has become a model for age-friendly work in higher education and discover practical strategies for fostering an inclusive and supportive environment for all ages.

